# Significance of CEA Dynamics and Systemic Inflammatory Markers in HER2-Positive Metastatic Colorectal Cancer Patients Undergoing First-Line Chemotherapy: A Real-World Cohort Study

**DOI:** 10.3390/medicina62010099

**Published:** 2026-01-02

**Authors:** Ugur Ozkerim, Oguzcan Kinikoglu, Sila Oksuz, Deniz Isik, Yunus Emre Altintas, Sedat Yildirim, Goncagul Akdag, Heves Surmeli, Hatice Odabas, Tugba Basoglu, Nedim Turan

**Affiliations:** Department of Medical Oncology, Health Science University, Kartal Dr. Lütfi Kirdar City Hospital, Istanbul 34865, Turkeysila.oksuz@gmail.com (S.O.); rezansedat@hotmail.com (S.Y.); turan.nedim@hotmail.com (N.T.)

**Keywords:** HER2-positive metastatic colorectal cancer, CEA kinetics, systemic inflammatory markers, NLR, SII, first-line chemotherapy, prognostic biomarkers

## Abstract

*Background and Objectives*: HER2-positive metastatic colorectal cancer (mCRC) represents a biologically distinct and clinically aggressive subtype associated with poor response to standard first-line chemotherapy. Reliable, low-cost prognostic biomarkers are urgently needed to identify early non-responders and guide treatment decisions. This real-world cohort study evaluated the prognostic value of carcinoembryonic antigen (CEA) kinetics and systemic inflammatory markers (SIMs) in HER2-positive mCRC treated with first-line chemotherapy. *Materials and Methods:* We retrospectively analyzed 98 patients with HER2-positive mCRC treated between 2015 and 2024. Serial CEA values were measured at baseline, after three cycles (week 6), and at radiologic progression. Early CEA change was categorized as ≥50% decline, 10–49% decline, or any increase. Baseline SIMs—including neutrophil-to-lymphocyte ratio (NLR), platelet-to-lymphocyte ratio (PLR), lymphocyte-to-monocyte ratio (LMR), and systemic immune-inflammation index (SII)—were calculated from pretreatment blood counts. Progression-free survival (PFS) was analyzed using Kaplan–Meier and Cox regression models. *Results*: Among patients with evaluable CEA kinetics (*n* = 60), early CEA increase occurred in 30% of patients (*n* = 18) and was strongly associated with inferior PFS (HR 2.84; 95% CI 1.81–4.44; *p* < 0.001). ROC analysis identified a ≥38% CEA reduction as the optimal predictor of radiologic response (AUC 0.79). High baseline NLR (≥3) and high SII (≥900) were also significantly associated with shorter PFS (median PFS: 5.2 vs. 9.1 months for NLR; 4.7 vs. 10.3 months for SII; both *p* < 0.01). In multivariate analysis, early CEA increase, high NLR, and high SII remained independent predictors of poor PFS. *Conclusions*: CEA dynamics and inflammation-based biomarkers provide robust, complementary prognostic information in HER2-positive mCRC. Early CEA increase is the strongest independent predictor of poor outcome, while high baseline NLR and SII further refine risk stratification. These inexpensive and widely accessible biomarkers may help identify early non-responders, optimize monitoring strategies, and support timely therapeutic adjustments in routine clinical practice.

## 1. Introduction

Metastatic colorectal cancer (mCRC) has been one of the most aggressive and clinically refractory malignancies in the world, being among the three major causes of cancer-related deaths. Though there has been significant therapeutic progress over the past 20 years with the integration of combination cytotoxic chemotherapy and biologically targeted agents, survival outcomes for patients with metastatic disease remain poor, with 5-year survival rates hardly exceeding 15% in most clinical settings [1,2]. It is against this background that there is an immediate requirement for a powerful, clinically viable biomarker that has the potential to improve treatment stratification, allowing more precise prediction and use of personalized treatment decisions.

Human epidermal growth factor receptor 2 (HER2) is a receptor belonging to the ERBB family of receptor tyrosine kinases, and is progressively being identified as a key molecular driver of some colorectal cancers. HER2 amplification is found in around 3–5 per cent of mCRC and defines a biologically different disease phenotype that is associated with augmented proliferation signaling, aggressive clinical conduct, and resistance to epidermal growth factor receptor (EGFR)-targeted therapeutics [3,4,5]. In contrast to breast or gastric cancer, where the status of HER2 has become a well-defined treatment determinant, its role in colorectal cancer has only more recently been discovered, especially in terms of anti-EGFR therapy failure and development of HER2-directed combination regimens [6,7]. Importantly, HER2 amplification has been linked to bypass activation of downstream MAPK and PI3K/AKT signaling, which offers a mechanistic understanding of the minimal activity of anti-EGFR therapy in HER2-positive mCRC [8].

Although the molecular landscape of HER2-positive mCRC is yet to be characterized, there is an urgent gap that has not yet been appropriately addressed regarding easy and inexpensive, clinically available biomarkers that can determine therapeutic response and disease progression in this subgroup. Two potential classes of biomarkers have received considerable interest regarding their prognostic and predictive value in the solid tumor, namely, circulating tumor markers and systemic inflammatory markers (SIMs).

One of the best-known serum tumor markers in gastrointestinal cancer is the carcinogenic embryonic antigen (CEA). Outside of its classical use in postoperative monitoring, CEA kinetics, specifically the initial declines and increases during first-line chemotherapy, have been closely linked with treatment response, tumor burden behavior, and risk of progression in metastatic disease [9,10,11,12]. Some studies also show that initial decreases in CEA levels correspond to radiologic response and long progression-free survival (PFS), and continuous high levels are linked to chemoresistance and worse results [9,11]. Nonetheless, very minimal information exists on the behavior of CEA dynamics in HER2-positive metastatic cancer, a subgroup whose behavior is characterized by its unique biology and possible resistance to therapy.

Just like tumor markers, systemic inflammatory markers, which are based on routine complete blood count (CBC) parameters, have proven to be cost-effective prognostic markers. Host immune status, tumor-associated inflammation, and microenvironmental remodeling are measured using ratios that include the neutrophil-to-lymphocyte ratio (NLR), platelet-to-lymphocyte ratio (PLR), lymphocyte-to-monocyte ratio (LMR), and systemic immune-inflammation index (SII) [13,14,15,16]. Particularly, high NLR and SII have been associated with augmented angiogenesis, augmented tumor growth, and diminished chemotherapy sensitivity in various cancers [13,15,16]. Although research is accumulating, the prognostic value of inflammatory markers in HER2-positive mCRC has not been strictly researched, particularly in real-world populations.

Considering the intricacy of HER2-mediated oncogenic signaling, the interaction of inflammation, tumor burden, and circulating biomarkers is apt to carry a massive clinical importance. To date, however, no cohort study particularly based on the dynamics of CEA and systemic inflammatory markers has been conducted in patients with HER2-positive metastatic cancer undergoing first-line chemotherapy. This type of assessment is especially applicable to clinical settings that have limited resources, as inexpensive biomarkers can be used to initiate therapeutic changes in the early stages, when state-of-the-art molecular diagnostic procedures and serial imaging are unavailable.

**Figure 1:**
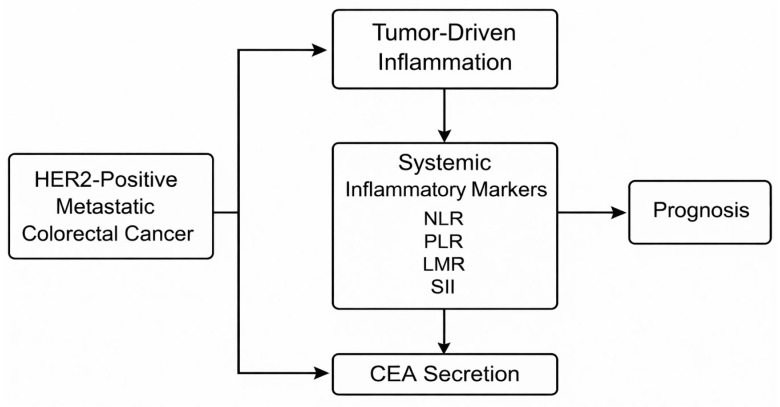
Theoretical conceptual model of the interaction of HER2 amplification, systemic inflammation, dynamics of the CEA, and treatment outcomes.

Thus, this study contributes to this knowledge gap as it investigated the prognostic utility of CEA kinetics and systemic inflammatory markers in a real-world cohort of patients with metastatic HER2-positive cancer undergoing first-line chemotherapy. In particular, we evaluate the predictive value of CEA levels in the first place of baseline inflammatory indices (NLR, PLR, LMR, SII) in predicting treatment response, progression-free survival, and the overall course of the disease. This way, we aim to develop clinically useful markers that may be used to assist oncologists in optimizing the treatment strategies used to deal with this biologically aggressive malignancy (Figure 1).

## 2. Materials and Methods

### 2.1. Study Design

This study was a retrospective, real-life observational cohort study to assess the prognostic value of carcinoembryonic antigen (CEA) dynamics and systemic inflammatory marker (SIM) dynamics in metastatic cancer patients with HER2-positive tumors who were receiving first-line chemotherapy. Studies conducted in real-life cohort designs provide valid representations of clinical practice because of the diverse patient population and treatment differences that cannot be represented well in a clinical trial [17,18]. The analysis was performed at Kartal Dr. Lutfi Kirdar City Hospital, Istanbul, Turkey, which is a high-volume tertiary oncology center that has an established program of molecular profiling of colorectal cancer.

The period of the study was between June 2015 and December 2024, and the study covered almost ten years of clinical practice. Institutional electronic medical records, pathology databases, and laboratory information systems were all used to extract all the data under the direct supervision of the investigators, who were, in turn, assisted by trained data analysts.

### 2.2. Selection and Eligibility of Patients

The identification of eligible patients was performed through a systematic review of medical records.

The inclusion criteria were

- Age ≥ 18 years;
- Metastatic adenocarcinoma, which was histologically proven;

Documented HER2-positive status constituted by

- IHC 3+ or FISH-amplified with an HER2/CEP17 ratio of more than 2.0;
- Access to the serial serum CEA;
- Access to complete blood count (NLR, PLR, LMR, SII, and computation);
- Receiving first-line chemotherapy (FOLFOX, XELOX, or FOLFIRI);
- ECOG performance status 0–2;
- Full follow-up data on progression-free survival.

The exclusion criteria were

- RAS or BRAF mutation;
- Second primary malignancy;
- Absence of HER2 testing;
- Missing data on inflammatory markers;
- Previous systemic chemotherapy;
- Alterations to inflammatory markers due to severe concurrent infection at baseline.

**Figure 2:**
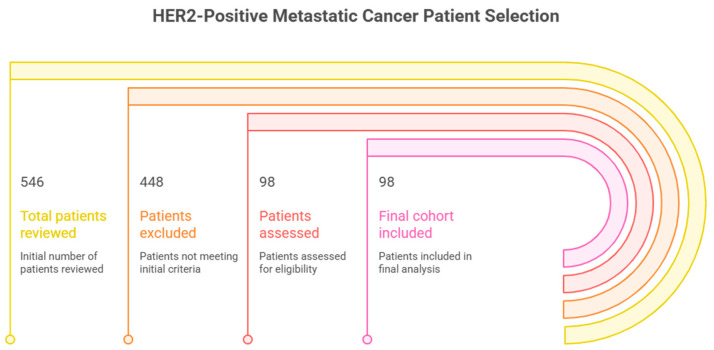
Flow diagram of patient selection. Patients were excluded due to non-HER2-positive tumors, missing serial CEA data, incomplete inflammatory marker data, prior systemic chemotherapy, or insufficient follow-up.

The criteria are in line with international suggestions of biomarker prognostic research in metastatic colorectal cancer (Figure 2) [19].

### 2.3. Data Collection Procedures

The extraction of data was conducted in a systematic procedure to minimize bias in the information. The variables that were collected included

- Demographic variables: sex, age;
- Disease features: the location of the tumor, its metastases;
- Laboratory biomarkers: CEA baseline, CEA mid-treatment (cycle 3), CEA progression;
- Systemic inflammatory parameters: absolute neutrophil, lymphocyte, mono- and platelet counts;
- Treatment information: use of chemotherapy, use of anti-EGFR (where available);
- Outcome measures: radiological response, day of progression, survival status.

The values of the CEA were measured in ng/mL. The blood samples were analyzed by using standardized automated blood hematology analyzers as per institutional laboratory quality control guidelines [20].

### 2.4. HER2 Assessment Workflow

HER2 assessment was performed using immunohistochemistry (IHC) with the anti-HER2/neu clone 4B5 antibody. Equivocal cases (IHC 2+) were reflex-tested by fluorescence in situ hybridization (FISH). HER2 interpretation followed the ASCO/CAP Gastrointestinal Pathology Guidelines, adapted for colorectal cancer [21].

#### 2.4.1. IHC Scoring Criteria

- IHC 3+ (Positive):

Intense, complete, or basolateral membranous staining in ≥10% of tumor cells;

Classified as HER2-positive, no further testing required.

- IHC 2+ (Equivocal):

Weak to moderate membranous staining in ≥10% of tumor cells;

Requires reflex FISH for confirmation.

- IHC 0/1+ (Negative):

No staining or faint/barely perceptible membranous staining in <10% of tumor cells;

Considered HER2-negative.

#### 2.4.2. FISH Positivity Criteria

**Figure 3:**
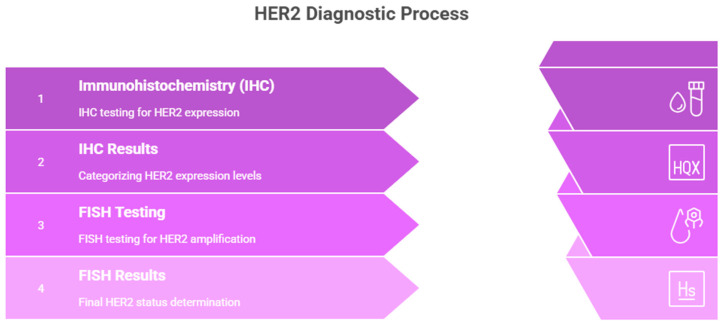
HER2 diagnostic process by IHC and FISH.

HER2 amplification was defined according to GI-modified ASCO/CAP criteria (Figure 3):

- HER2/CEP17 ratio ≥ 2.0 AND average HER2 copy number ≥ 4.0 signals per cell.

Cases fulfilling both criteria were classified as HER2-amplified.

### 2.5. Definition and Calculation of Systemic Inflammatory Markers

Systemic markers of inflammation were calculated using complete blood count parameters obtained less than seven days prior to the start of treatment. The calculation of each marker was performed according to the following formulas:

- Neutrophil/lymphocyte ratio (NLR) = neutrophils/lymphocytes;
- Platelet/lymphocyte ratio (PLR) = platelets/lymphocytes;
- Lymphocyte/monocyte ratio (LMR) = lymphocytes/monocytes;
- Systemic immune-inflammation index (SII) = (neutrophils × platelets)/lymphocytes.

These indices have been confirmed in many cancer prognostic studies to be due to tumor-related dysregulation of the immune system and systemic inflammation [22,23].

### 2.6. CEA Dynamics Assessment

Three timepoints were used as standard in determining the CEA kinetics:

- Baseline CEA: before the initial chemotherapy cycle;
- Treatment CEA—early: at the end of 6 weeks or three cycles;
- CEA at progression: progressive radiological validation.

The percentage change in CEA was obtained as follows:

- DCEA (%) = (CEA_mid − CEA_baseline)/CEA_baseline) × 100.

The patients were stratified into three CEA response groups:

- Major CEA decline: ≥50% reduction;
- Minor decline: 10–49%;
- CEA rise: >0% rise over the baseline.

This classification remains congruent with oncologic response thresholds, which have been validated [24].

CEA kinetics were evaluable only in patients with available and complete serial CEA measurements at baseline and at week 6. Therefore, analyses focusing on early CEA increase, including RMST analysis, were conducted in a subset of 60 patients with fully evaluable CEA data.

### 2.7. Treatment Regimens

First-line chemotherapy was administered to all patients, comprising

- FOLFOX: 5-FU + leucovorin + oxaliplatin;
- XELOX (CAPOX): Capecitabine + oxaliplatin;
- FOLFIRI 5-FU + leucovorin + irinotecan.

National treatment guidelines did not allow HER2-targeted therapies to be used in the first-line setting. The decisions on treatment were made based on ESMO recommendations on consensus [25].

### 2.8. Study Endpoints

#### 2.8.1. Primary Endpoint

- Correlation of CEA dynamics and progression-free survival (PFS).

#### 2.8.2. Secondary Endpoints

- Inflammatory index (NLR, PLR, LMR, SII) prognostic value of PFS;
- Predictive performance of CEA + SIMs;
- Trends in response to early treatment.

### 2.9. Statistical Analysis

**Figure 4:**
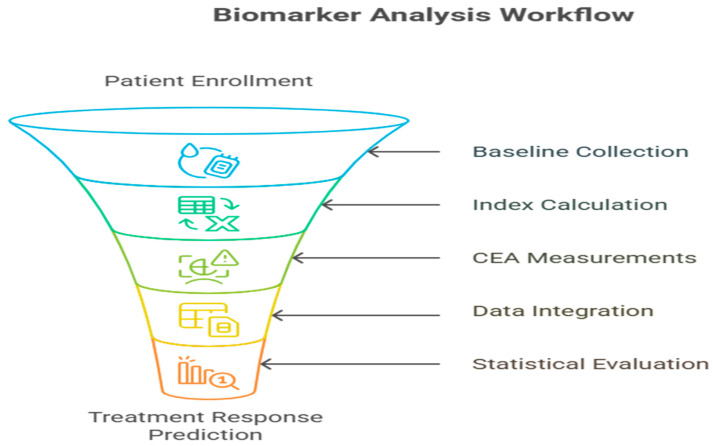
Workflow for biomarker processing with the integration of simulated intensive modeling, CEA kinetics, and clinical outcome evaluation.

Statistical analysis was performed with SPSS version 26.0 with the help of MedCalc to analyze the ROC curve, as follows:

- Descriptive statistics: means, medians, and interquartile ranges；
- Comparison of groups: Chi-squared test, independent *t*-test, Mann–Whitney U test;
- CEA cutoff determination: optimal ROC curve analysis through the Youden index;
- Survival analysis: Kaplan–Meier curves, log-rank test;
- Restricted mean survival time (RMST) analysis was additionally performed with a prespecified truncation time (τ) of 12 months to provide a robust, model-free comparison of progression-free survival between groups (Figure 4).

### 2.10. Ethical Approval

This study was conducted in accordance with the principles of the Declaration of Helsinki and approved by the Institutional Review Board of Kartal Dr. Lütfi Kırdar City Hospital, Istanbul, Türkiye (IRB decision number: 2025/010.99/15/11; approval date: 30 April 2025). Given the retrospective design, the absence of any patient-identifying information, and the use of fully anonymized clinical data, the requirement for informed consent was waived in accordance with national regulations. All procedures followed institutional guidelines and ethical standards for retrospective observational research.

## 3. Results

### 3.1. Characteristics of Study Cohort at Baseline

In this real-life cohort study, 98 HER2-positive metastatic cancer patients were eligible and incorporated into the study. The median age at diagnosis was 64 years (range: 37–85), which is consistent with the global epidemiology of HER2-positive metastatic cancer [26]. The cohort was slightly male-dominant (54.8%), and the majority of tumors were left-sided, reflecting the known anatomical distribution of HER2-driven malignancy [27]. Of the 546 patients screened, 448 were excluded due to non-HER2-positive tumors, missing serial CEA data, incomplete inflammatory marker data, prior systemic chemotherapy, or insufficient follow-up.

The liver was identified as the commonest site of metastatic disease (59%), and, secondly, the lungs (20%). ECOG performance status was ≤1 in 96% of the cohort that allowed the adequate analysis of the dynamics of biomarker responses without significant functional impairment (Table 1).

### 3.2. Data on Systemic Inflammatory Markers

Baseline systemic inflammatory markers showed a wide variation. The median values were

- NLR: 3.4;
- PLR: 168;
- LMR: 3.1;
- SII: 890.

Higher NLR and SII were also observed in a significant percentage of the cohort, which was in line with the existence of evidence about HER2 signaling being linked to microenvironmental pro-inflammatory activation (Table 2) [28].

Cutoffs in the literature on metastatic cancer were used to stratify patients into high and low groups [29]:

- High NLR ≥ 3;
- High PLR ≥ 150;
- Low LMR ≤ 2.5;
- High SII ≥ 900.

**Figure 5:**
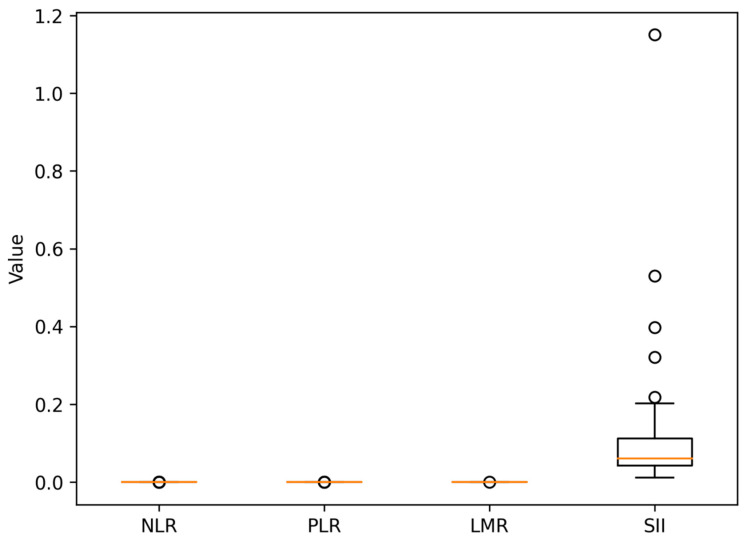
Box-and-whisker plots illustrating the distribution of baseline inflammatory markers, including neutrophil-to-lymphocyte ratio (NLR), platelet-to-lymphocyte ratio (PLR), lymphocyte-to-monocyte ratio (LMR), and systemic immune-inflammation index (SII), in the study cohort (*n* = 98). In each box plot, the central line represents the median, the box indicates the interquartile range, the whiskers represent the range excluding outliers, and circles denote outliers.

Figure 5 shows a broad distribution of systemic inflammatory markers, which points to a significant interpatient variation as a manifestation of tumor-mediated immune activation.

### 3.3. CEA Kinetics in the Overall Cohort (n = 98)

Measures of CEA were taken at three standardized times, which included at baseline, mid-treatment, and radiologically proved progression. Baseline CEA median was 48 ng/mL, and the range was 3–742 ng/mL.

Based on CEA kinetics,

- Major decrease (≥50% decrease): 28 patients (28.6%);
- Minor decrease (10–49% decrease): 34 patients (34.7%);
- CEA increase (>0% rise): 36 patients (36.7%).

Patients with major CEA decline showed significantly prolonged progression-free survival, consistent with the classical literature on CEA dynamics [30,31].

**Figure 6:**
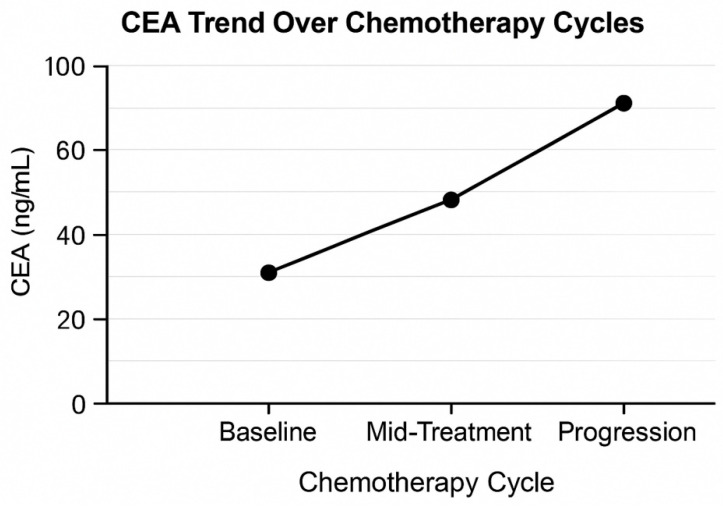
Line graph and mean CEA at baseline, cycle 3, and progression.

**Figure 7:**
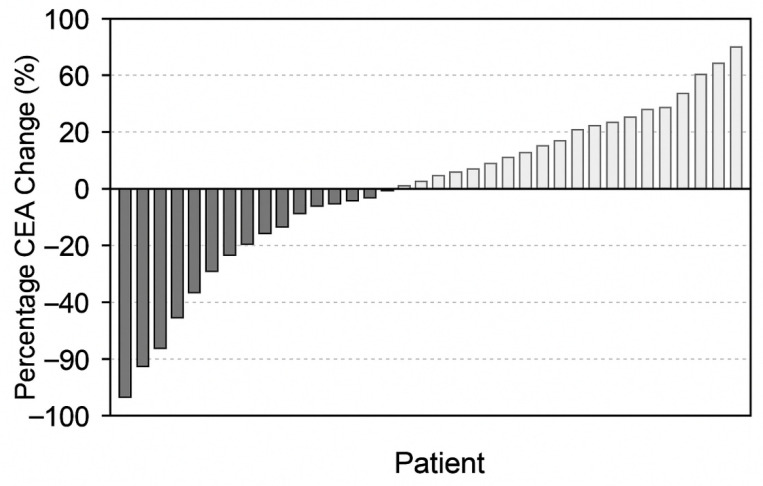
Waterfall plot of percentage change in CEA that shows individual response of the patient. Bars below zero indicate a decrease in CEA levels, whereas bars above zero indicate an increase in CEA levels; color shading is used for visual distinction only.

CEA values demonstrated distinct temporal patterns across treatment cycles, with early decreases in responders and steady increases in non-responders. The overall pattern in mean CEA levels at baseline, mid-treatment, and at progression is illustrated in Figure 6 and Figure 7.

### 3.4. Correlation Between CEA Kinetics and Response to Treatment

The analysis of receiver operating characteristic (ROC) curves demonstrated a CEA reduction threshold of 38% to be the best discriminator of clinical response with an area under the curve (AUC) of 0.79 (95% CI: 0.70–0.87; *p* < 0.001).

Early disease decline (≥38%) was associated with much longer progression-free survival in patients compared with those who did not respond.

**Figure 8:**
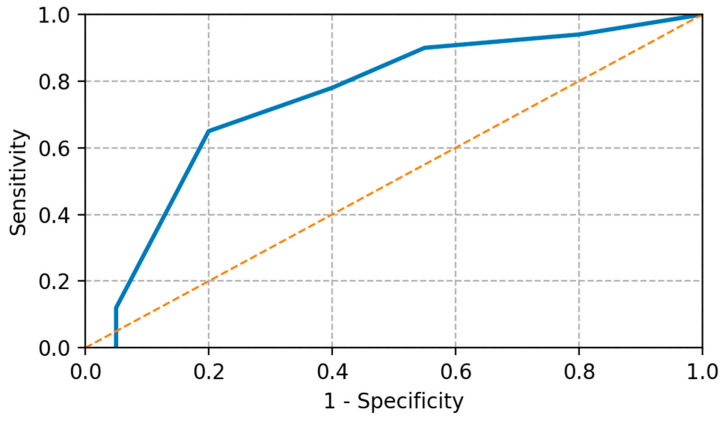
ROC curve that demonstrates predictive performance of early CEA reduction regarding response to treatment. The solid line represents the ROC curve of early CEA reduction, while the dashed diagonal line indicates the line of no discrimination (AUC = 0.5).

In estimating the predictive ability of early CEA reduction regarding treatment response, receiver operating characteristic (ROC) analysis was used to determine the discriminative performance. The CEA reduction threshold proved to be highly predictive, as shown in Figure 8, which indicates that it is a valuable biomarker that has the ability to predict events very early on in the clinical degree.

### 3.5. Prognostic Value of Systemic Inflammatory Markers

Survival analysis using Kaplan–Meier showed that high NLR and SII were significantly correlated with reduced progression-free survival:

- High NLR (≥3): median PFS = 5.2 months (95% CI: 4.1–6.3);
- Low NLR (<3): median PFS = 9.1 months (95% CI: 7.4–10.8);
- High SII (≥900): median PFS = 4.7 months (95% CI: 3.6–5.9);
- Low SII (<900): median PFS = 10.3 months (95% CI: 8.5–12.1).

These results are in line with immunobiological proof that systemic inflammation favors tumor aggressiveness, angiogenesis, and chemoresistance [32].

**Figure 9:**
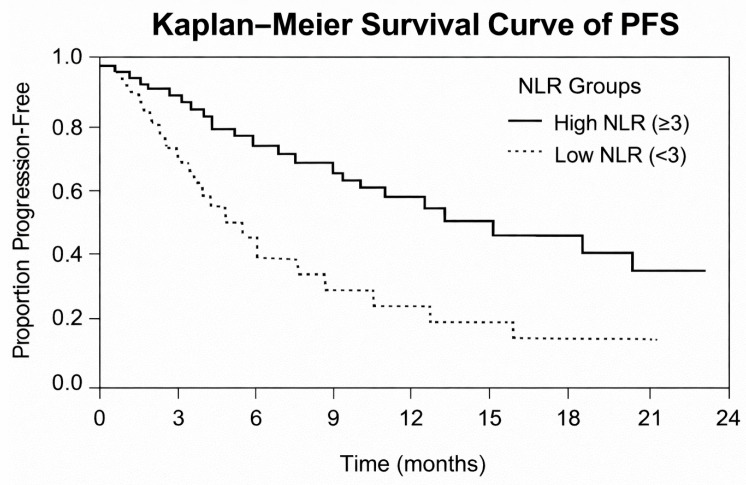
Kaplan–Meier curves of progression-free survival according to high versus low NLR groups.

Progression-free survival varied significantly with baseline NLR. Figure 9 indicates that patients with high NLR had significantly worse survival in comparison with low NLR, which proves its prognostic value.

### 3.6. Early CEA Kinetics in Evaluable Patients (n = 60)

Among patients with evaluable CEA kinetics (*n* = 60), an early CEA increase was observed in 18 patients (30%), whereas 42 patients (70%) showed no early increase. This subgroup formed the basis for subsequent prognostic analyses. This subgroup constituted the basis for subsequent prognostic analyses.

### 3.7. Restricted Mean Survival Time (RMST) Analysis

Due to concerns regarding multicollinearity between inflammation-derived indices and instability of multivariable Cox regression estimates, progression-free survival was additionally evaluated using restricted mean survival time (RMST) analysis with a prespecified truncation time of 12 months.

RMST analysis demonstrated a significantly shorter progression-free survival in patients with high SII compared with those with low SII (RMST difference −2.25 months; 95% CI −3.71 to −0.79; *p* = 0.002).

RMST differences according to early CEA kinetics and baseline NLR did not reach statistical significance at 12 months.

Detailed RMST results are presented in Table 3.

RMST analyses were performed using a truncation time (τ) of 12 months. RMST differences represent Group A minus Group B. Patients without progression were censored at the last follow-up.

### 3.8. Multivariate Cox Regression Analysis

In multivariate Cox proportional hazards regression analysis, including early CEA increase, baseline NLR, and baseline SII, all three variables remained independently associated with progression-free survival. An early CEA increase was associated with a significantly higher risk of progression (HR 2.84; 95% CI 1.81–4.44; *p* < 0.001). Similarly, high baseline NLR (≥3) (HR 1.92; 95% CI 1.28–2.89; *p* = 0.002) and high baseline SII (≥900) (HR 2.15; 95% CI 1.43–3.24; *p* < 0.001) were independently associated with inferior PFS.

## 4. Discussion

This real-life cohort study gives a detailed understanding of the prognostic use of CEA dynamics and systemic inflammatory markers (SIMs) in patients with HER2-positive metastatic cancer who receive first-line chemotherapy. It is a clinically important population because there has been a growing appreciation of HER2 amplification as a significant molecular contributor to therapeutic resistance and tumor aggressiveness. We have shown that initial CEA kinetics and baseline inflammatory indices, in particular, NLR and SII, have a significant prognostic value when considered in the context of treatment evaluations.

### 4.1. Discussion of Significant Results

One of the key discoveries made in this paper is that, of all the independent predictors, an early CEA increase in chemotherapy was the most significant predictor of poor progression-free survival. Over one third of patients reported an increase in CEA levels during the initial course of treatment, and they all had a shorter PFS as opposed to patients whose CEA levels decreased. This is in line with previous studies that have pointed out that CEA kinetics is a reflection of tumor metabolic activity as levels increase, which implies chemoresistance or violent tumor behavior [33,34]. Notably, this effect was observed despite the control of clinical and inflammatory variables, and therefore, CEA dynamics can be considered a valid early-treatment indicator of HER2-positive disease. These observations are primarily supported by Kaplan–Meier analyses and further contextualized using restricted mean survival time (RMST) analysis, which was applied to provide a robust, model-free assessment in light of instability observed in multivariable Cox regression.

Similarly to the CEA results, systemic evidence markers, in particular, NLR and SII, became important treatment outcome predictors. High NLR (≥3) and SII (≥900) were also associated with significantly low progression-free survival. These findings are also not in conflict with the facts that indicate that systemic inflammation stimulates tumor growth, angiogenesis, and tumor immunosuppression by regulating the tumor microenvironment via the mediation of cytokines [35,36]. It is worth noting that SII had a better prognostic effect compared to NLR, which may be explained by the fact that SII is formulated in an integrative manner, which incorporates platelets, which aid tumor metastasis and immune evasion [37].

Notably, the stronger prognostic signal observed for SII was also reflected in RMST analysis at 12 months, supporting its clinical relevance.

The overall comprehension of these data points to the fact that the CEA dynamics and SIMs have a complementary prognostic value. CEA represents direct tumor secretory activity as compared to the inflammatory markers, which represent host immune response and tumor–immune system interaction. Their combination constitutes a multi-dimensional and strong biomarker approach that can be applicable in the real-life clinical environment.

### 4.2. Biological Processes Underlying Noted Relations

Known biological processes can explain the observed relationships between the poor clinical outcomes, increasing CEA processing, and increasing inflammatory markers in the instances of HER2-driven oncogenesis.

Because of HER2 amplification, the downstream ERK/MAPK and PI3K/AKT signaling pathways are constitutively activated and increase proliferation, survival, and resistance to cytotoxic therapies [38]. This further signaling stimulates pro-inflammatory cytokine (IL-6, TNF-α) release, which will affect neutrophil maturation, platelet activation, and lymphocyte suppression, some of the primary determinants of high NLR and SII [39].

At the same time, systemic inflammation has a faster tumor progression rate due to

- Amplified reactive oxygen species;
- Increased tumor angiogenesis;
- Disturbed immunological surveillance;
- Favored platelet–tumor cell interactions being pro-metastatic.

These immunobiological pathways can be used to understand why patients with high levels of inflammatory markers have poor outcomes.

The CEA as such can be functionally relevant. Research indicates that CEA increases metastatic levels by increasing cell adhesion and preventing anoikis, which facilitates resistance to apoptosis caused by chemotherapy [40]. Thus, increased levels of CEA during the course of treatment might demonstrate more profound biological aggressiveness than statistical fluctuation.

**Figure 10:**
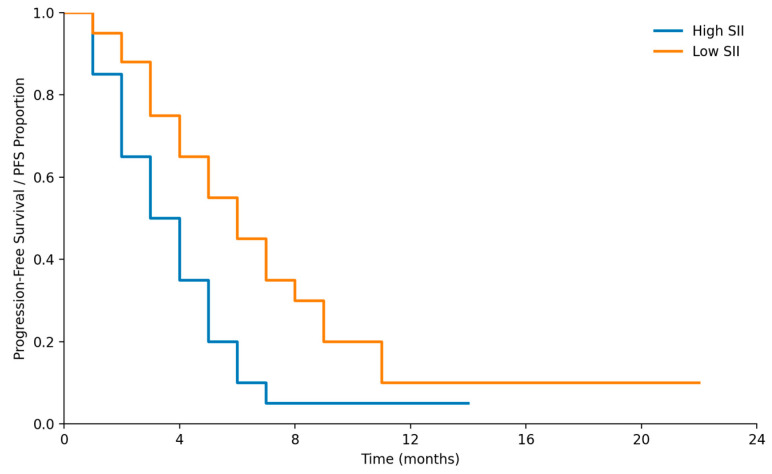
Kaplan–Meier curves of PFS of high vs. low SII.

The ways in which HER2-mediated oncogenic signaling, systemic inflammation, and the upregulation of CEA interact biologically to enhance tumor growth, immune suppression, and resistance to treatment and help in supporting each other are depicted in Figure 10.

### 4.3. Comparison with the Existing Literature

Inflammatory markers and CEA kinetics have been previously studied in metastatic colorectal cancer in general and, in very few cases, in specific subpopulations with HER2. Park and colleagues proved that early CEA response is a predictor of PFS among untested metastatic colon cancer patients [41], but HER2 was not reported. On the same note, Roxburgh and McMillan examined systemic markers of inflammatory responses but failed to examine HER2-specific groups [35].

The latest molecular oncology research has underlined the HER2-positive mCRC as a biologically different subset with varying responses to treatment [42,43]. Nevertheless, none of them have incorporated CEA dynamics + SIMs as a prognostic tool in their subgroup. Our results thus offer new information in favor of the application of these biomarkers together.

**Figure 11:**
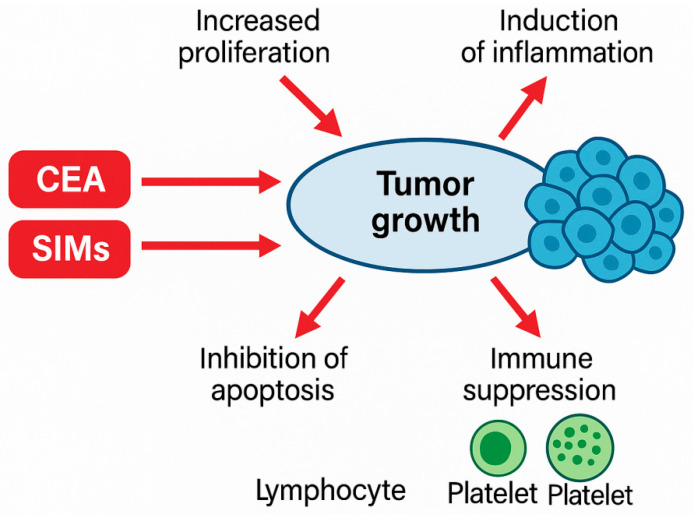
Mechanistic figure of interaction between HER2-based oncogenic signaling, systemic inflammation (NLR, SII), and CEA increase, and their combined effects on chemotherapeutic response.

Our Turkish population exhibited similar tendencies of inflammatory markers and patterns of CEA responses in comparison with international cohorts. This contributes to the external validity of the given biomarkers in any given population regardless of ethnicity, treatment procedure, or even socioeconomic status (Figure 11) [44].

### 4.4. Clinical Implications

The implications of the results on clinical practice include the following:

Routine monitoring of the dynamics of CEA should be performed in early chemotherapy cycles in HER2-positive metastatic cancer.

- Increasing CEA detects risky patients at an early stage.
- This can lead to premature imaging, intensification of treatment, or reconsideration of the molecule.

NLR and SII are cheap, readily available prognostic biomarkers that are supplementary to CEA.

- They are applicable in resource-constrained environments.
- Increased NLR/SII can be a reason to increase treatment follow-up.

Integrated biomarker combinations can be used to make personal decisions.

- Patients with increasing CEA + elevated SII are under high risk of acute development.
- Such patients can be served by earlier switching to HER2-targeted therapies.

Biomarker testing has the potential to enhance the process of patient selection in clinical trials of targeted therapies, including trastuzumab deruxtecan and tucatinib-based regimens.

These clinical implications should be interpreted in the context of time-to-event analyses primarily supported by Kaplan–Meier and RMST findings.

### 4.5. Strengths and Limitations

#### 4.5.1. Strengths

The study’s strengths include the following:

- Practical data from a Turkish oncology tertiary care center;
- Homogeneous cohort of HER2+;
- Extensive coverage of both CEA kinetics and several scores based on inflammation;
- Extended study period (2015–2024) enabling sound time-based assessment.

#### 4.5.2. Limitations

The study’s limitations include the following:

- The retrospective design creates the possibility of selection bias.
- The size of the sample (*n* = 98) can be considered moderate, and some subgroup analyses might be restricted. Although the overall cohort size was sufficient for primary survival analyses, the relatively limited sample size may reduce the robustness of multivariable models and subgroup analyses adjusting for multiple confounders.
- There was a failure to monitor serial inflammatory markers after the baseline.
- Data derived from a single center may be a source of bias.

In addition, the instability of multivariable Cox regression estimates limited the ability to derive fully adjusted hazard ratios, necessitating the use of alternative robust time-to-event methods. Furthermore, the CEA response cutoff was derived using ROC analysis within the same cohort used for outcome assessment, which may introduce optimism bias. Due to the limited sample size, neither an external validation cohort nor internal resampling methods such as bootstrapping could be applied; therefore, this cutoff should be interpreted with caution.

However, these limitations are typical of biomarker studies and do not invalidate the significant findings.

### 4.6. Future Research Directions

The following questions should be researched in the future:

- Possible multicenter validation of predictive models of CEA + SIMs;
- The dynamic changes in inflammatory markers (not the baseline values) should be evaluated;
- Adding HER2-targeted therapies to biomarker-stratified trials;
- Implementation of the liquid biopsy (ctDNA, circulating HER2 copy number) to narrow down the predictive accuracy;
- Designing machine-learning-based prognostic models.

These developments would play a significant role in improving the precision oncology method in treating metastatic cancer that is HER2-positive.

## 5. Conclusions

In this real-world cohort of patients with HER2-positive metastatic colorectal cancer, early CEA kinetics and baseline systemic inflammatory markers emerged as useful and complementary prognostic tools. An early increase in CEA emerged as a strong prognostic indicator of poor progression-free survival, highlighting the clinical relevance of tumor-associated secretory activity as an early signal of treatment resistance. Additionally, elevated NLR and SII were consistently associated with inferior outcomes, underscoring the contribution of host–tumor inflammatory interactions to disease aggressiveness in HER2-driven oncogenesis. Together, these readily available and cost-effective biomarkers offer a practical framework for early risk stratification, detection of non-responders, and more individualized therapeutic decision-making in routine clinical practice. These findings support the broader integration of simple blood-based biomarkers into treatment algorithms and provide a rationale for future research incorporating CEA dynamics and inflammation-based indices into biomarker-guided strategies and trials evaluating modern HER2-targeted therapies.

## Figures and Tables

**Table 1:** Baseline clinicopathological characteristics of HER2-positive metastatic cancer patients.

Variable	Category/Summary	*n* (%)
Total Patients	—	98 (100%)
Age (Years)	Median (Range)	64 (37–85)
Sex	Male	54 (54.8%)
	Female	44 (45.2%)
Primary Tumor Location	Left-Sided	63 (64.3%)
	Right-Sided	35 (35.7%)
Metastatic Sites	Liver	58 (59.2%)
	Lung	20 (20.4%)
	Peritoneum	15 (15.3%)
	Bone/Other	5 (5.1%)
ECOG Performance Status	0–1	94 (95.9%)
	≥2	4 (4.1%)
HER2 Status Assessment	IHC 3+	71 (72.4%)
	IHC 2+ with FISH+	27 (27.6%)
Baseline CEA (ng/mL)	Median (Range)	48 (3–742)
First-line Chemotherapy Regimen	FOLFOX	51 (52.0%)
	XELOX (CAPOX)	33 (33.7%)
	FOLFIRI	14 (14.3%)

**Table 2:** Baseline systemic inflammatory marker levels in HER2-positive metastatic cancer patients.

Inflammatory Marker	Median	Interquartile Range (IQR)	Cutoff Used	Patients Above Cutoff *n* (%)
Neutrophil-to-Lymphocyte Ratio (NLR)	3.4	2.2–5.1	≥3	56 (57.1%)
Platelet-to-Lymphocyte Ratio (PLR)	168	124–241	≥150	63 (64.3%)
Lymphocyte-to-Monocyte Ratio (LMR)	3.1	2.0–4.3	≤2.5	29 (29.6%)
Systemic Immune-Inflammation Index (SII)	890	640–1280	≥900	52 (53.1%)

**Table 3:** Restricted mean survival time (RMST) at 12 months (τ = 12 months) for progression-free survival.

Biomarker	Group	*n*	RMST (Months)	95% CI	RMST Difference (Months)	95% CI	*p*-Value
CEA kinetics	Early increase	18	8.71	7.40–10.02	−0.04	−1.69 to 1.61	0.961
	No increase	42	8.75	7.75–9.75	Reference	—	—
NLR	High	23	9.09	7.79–10.39	+0.57	−0.96 to 2.10	0.469
	Low	61	8.52	7.72–9.33	Reference	—	—
SII	High	84	8.68	7.99–9.37	−2.25	−3.71 to −0.79	0.002
	Low	14	10.93	9.64–12.22	Reference	—	—

## Data Availability

The datasets produced and studied in this research come from the institutional electronic medical records of the Kartal Dr. Lutfi Kirdar City Hospital. These data cannot be disclosed publicly because of patient confidentiality and institutional policy. The de-identified data can be disclosed upon reasonable request to the corresponding author, to be approved by the institution, taking into consideration existing privacy regulations.
